# A Case of Tic Douloureux

**Published:** 1821-11

**Authors:** Benjamin Hutchinson

**Affiliations:** Member of the Royal College of Surgeons.


### *A Case of Tic Douloureux.* By Benjamin Hutchinson, Member of the Royal College of Surgeons.

THOMAS NEEP, the subject of the following case, resides at Southwell, in the county of Nottingham, aged fifty-five years; a man of delicate fibre, with evident traces of scrofulous diathesis; and in the former part of his life he was much addicted to intemperate habits; by trade a hawker. He has been subject to the tortures of tic douloureux during the last ten years. The primary symptoms of the disease were preceded by, and accompanied with, considerable torpor and drowsiness; to remove which, the surgeon, whom he had consulted, had recourse to the judicious means of topical bleeding with leeches, of purging to a great extent, and to other measures suggested by discrimination and skill. This unusual lethargic disposition continued with little abatement for the space of two years; the torments in his face, at the same time, resisting every well-directed remedy for their relief. The facial pains, at their commencement, were moderate, but became more violent in their progress; and at length acquired that exquisite acuteness, which neither the imagination can readily conceive, nor words easily describe. The paroxysms generally began in the upper gums, extending upwards under the eye, diverging towards the alæ nasi, and the whole of the right side of the face. The pain was not of the continued obtuse kind, like that of chronic rheumatism; but, on the contrary, rather transient, exceedingly acute and lancinating during its attack. The periods of its recurrence were indefinite, in the intervals of which he was in comparative ease. There was some uniformity in the direction and origin of the pain: it always began in the gums and upper lip, and darted upwards towards the orbit; the same sensations were also observed on the bony and fleshy palates, on the gums and teeth of the upper jaw, and sometimes on the fauces. Not any exemption from his sufferings could be expected from changes of seasons or of climate; though windy weather seemed to increase the complaint to so intense a degree as to prevent him using the exercise necessary in pursuing his ordinary business. At different periods, my patient suffered the extraction of nine sound teeth, without experiencing the slightest relief: on the contrary, after the removal of each tooth, he endured a perceptible increase in the number and violence of painful paroxysms. He would sometimes suffer five or six of these pains in an hour, while at others he would only have as many in a day; and, of course, they sensibly varied in their degree of violence. The disease produced no perceptible change in the size or colour of the cheek, excepting what might be attributed to the application of external rubefacients. His sufferings compelled him to have recourse to every variety of means which held out a promise of relief; all of which, however, were equally unavailing. Topical and general bleeding, blistering the part affected, the internal use of the remedies so frequently mentioned in my former narratives, were alike inefficacious. The disease appears to have had its immediate seat in the second branch of the fifth pair of nerves, the superior maxillary, which, passing through the foramen rotundum, distributes its branches to those parts which I have endeavoured to describe as being principally affected. As at times my patient suffered also, during the paroxysms, from a copious effusion of scalding tears, that branch of the ophthalmic which is distributed to the lachrymal gland was probably in a state of morbid irritation. The portio dura of the seventh pair, the pes anserinus, sympathized also with this direful nervous derangement; its branches communicating with several of those of the fifth pair.

In the month of March last, Thomas Neep was recommended to put himself under my management; when he immediately began to take one drachm of the carbonate of iron twice a-day, mixed in honey; and to make use of the emetic-tartar ointment, with powdered opium and camphor, applied in a certain quantity to his face every night. After a patient trial of five weeks in these means, his pains were something alleviated: the medicine, however, produced considerable diarrhœa, which was restrained by the addition of a few drops of the tincture opii. His torments, though considerably mitigated, were still almost intolerable, and I increased the quantity of the carbonate of iron to four scruples three times a-day, in which quantity he persevered for two months, at which period the violence of the disease began evidently to yield to the influence of the medicine; and, after a perseverance in this increased dose for three months, with some few and short intervals, his pains left him.

I have seen him this morning, (August 28th,) when he favoured me with the outlines of this history; and he declares that his comforts and feelings are too great for his powers of description. Occasionally, though but seldom, he has very slight sensations, reminding him of his past sufferings: nothing, however, which he regards, or has the most remote care about.

*Southwell; 1st Sept.* 1821.

